# Leveraging single B cell antibody platforms to develop countermeasures against animal viral diseases: recent advances and future perspectives

**DOI:** 10.3389/fvets.2026.1854176

**Published:** 2026-05-26

**Authors:** Pengfei Shi, Zheya Zhang, Weimei He, Yu Duan, Yulong He, Huapeng Feng, Jianhong Shu

**Affiliations:** 1 College of Life Sciences and Medicine, Zhejang Sci-Tech University, Hangzhou, China; 2 Hangzhou ISEEVAX Medical Sciences and Technology, Co., Ltd., Hangzhou, China

**Keywords:** monoclonal antibody, single B-cell technology, veterinary, virus, zoonotic

## Abstract

The discovery and application of monoclonal antibodies (mAbs) have fundamentally revolutionized clinical medicine, driving significant paradigm shifts in disease prevention, precise diagnosis and targeted treatment. The demand for high-affinity antibodies continues to surge, yet traditional discovery platforms such as hybridoma and phage display suffer from low efficiency, disrupted native pairing, and time-consuming workflows. Single B cell antibody technology overcomes these limitations by directly retrieving naturally paired heavy and light chain sequences from antigen-specific B cells, thereby preserving authentic affinity and specificity. Following its remarkable success in combating human emergencies like COVID-19, this platform is now being rapidly adapted to veterinary viral diseases, a critical effort under the “One Health” framework to safeguard both animal and public health. This review systematically outlines the core principles and workflows of single B cell-based mAb discovery. It then summarizes recent advances in applying this technology to viruses that infect animals and zoonotic viruses, emphasizing how native antibody repertoires have been harnessed to generate high-affinity mAbs. Ultimately, the article concludes with a discussion of current technical challenges, proposing future research directions to improve both the accessibility and efficacy of veterinary immunotherapeutics.

## 1 Introduction

Infectious diseases remain a persistent and critical threat to global health, significantly impacting both human and animal populations. Among these, viral infections constitute a particularly formidable category, distinguished by their high pathogenicity, rapid genomic mutation, and capacity for immunosuppression (1). Indeed, viruses account for a substantial proportion of emerging infectious diseases, as evidenced by the global outbreaks of dengue virus (DENV), severe acute respiratory syndrome coronavirus 2 (SARS-CoV-2), and Zika virus (ZIKV) (2–4). In the veterinary sector, the impact of viral pathogens is equally devastating, threatening global food security and animal welfare. Transboundary diseases such as African swine fever virus (ASFV) and foot-and-mouth disease virus (FMDV) cause catastrophic economic losses to livestock production, while pathogens like canine distemper virus (CDV) continue to endanger companion animals (5–7). Consequently, the development of precise diagnostic tools and effective therapeutic interventions has become an urgent priority in veterinary medicine. In response to these challenges, mAbs have emerged as premier biological products, offering high specificity, immediate onset of action, and viral neutralization. Concurrently, recent strides in protein engineering have expanded the application scope of mAbs, enabling the design of novel formats with enhanced pharmacokinetic profiles, optimized effector functions, and bispecific capabilities (8). The generation of mAbs has historically been constrained by technical limitations. Hybridoma technology, a traditional platform, possesses inherent drawbacks such as low fusion efficiency (9). Phage display, another traditional platform, also exhibits limitations including artificial heavy-light chain pairing and loss of native affinity (10). To overcome these limitations, the generation of mAbs has been accelerated by advanced methodologies, notably single B cell antibody technology. This approach represents a transformative strategy in the landscape of therapeutic discovery. By utilizing fluorescence-activated cell sorting (FACS) and microfluidics, researchers can directly interrogate the native immune repertoire (11). This capability allows for the rapid capture of rare, ultra-potent clones that might be lost during the inefficient hybridoma fusion process, compressing discovery timelines from months to mere weeks.

Strategies for mAb development in veterinary medicine have largely paralleled advancements in human biomedical research, yet they face unique challenges regarding genomic annotation and production costs. This review systematically clarifies the principles and workflows of single B cell-based technologies for the discovery of mAbs targeting viral pathogens in animals. It summarizes the applications of single B cell platform in major veterinary species. Ultimately, we examine the technical barriers to implementing single B cell technology in veterinary science and propose directions for future development to bridge the gap between academic innovation and clinical application.

## 2 Technological platforms for mAb isolation: from hybridoma to single B cell technologies

### 2.1 Mouse hybridoma technology

**Figure 1:**
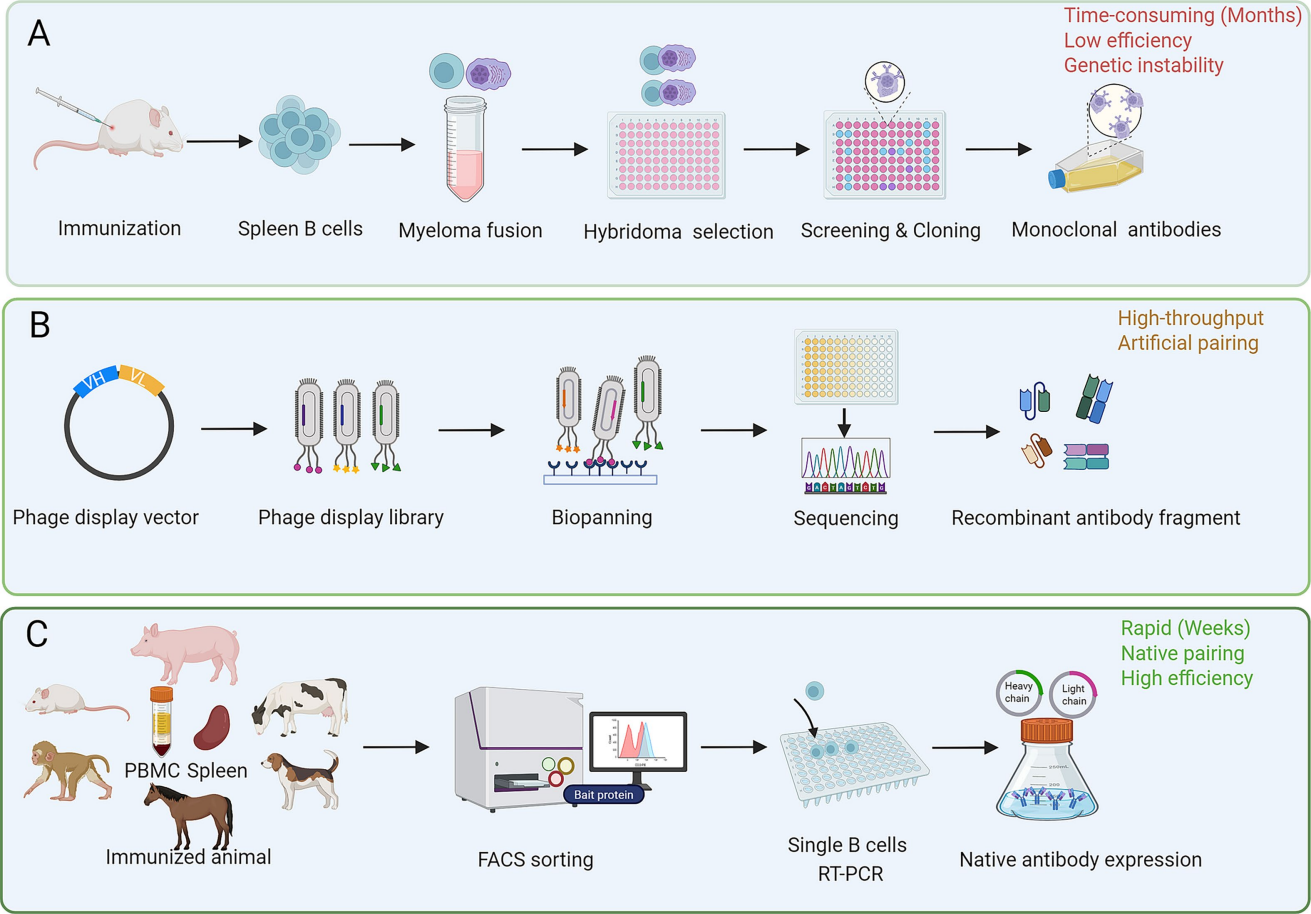
Comparative analysis of monoclonal antibody discovery platforms. **(A)** Traditional hybridoma technology relies on cell fusion, often resulting in low efficiency and loss of rare clones. **(B)** Phage display technology allows for high-throughput screening but may result in artificial heavy-light chain pairing. **(C)** Single B cell technology enables the direct isolation of native, antigen-specific antibody sequences, preserving natural affinity and reducing the discovery timeline from months to weeks.

Hybridoma technology, pioneered by Milstein and Köhler (12), relies on fusing antigen-primed B lymphocytes with immortalized myeloma cells to generate mAb secreting hybridomas (Figure 1A). Within the veterinary field, hybridoma technology has been extensively utilized to generate mAbs for major swine pathogens, including porcine epidemic diarrhea virus (PEDV), ASFV, and porcine reproductive and respiratory syndrome virus (PRRSV). These antibodies are integral to various diagnostic platforms, such as enzyme-linked immunosorbent assays (ELISA), immunofluorescence assays (IFA), and immunohistochemistry (IHC) (13–15). Hybridoma-derived mAbs are frequently conjugated with colloidal gold to facilitate point-of-care diagnostics, such as rapid test strips for detecting viral infections in companion animals (16). Despite its widespread utility, hybridoma technology is constrained by several inherent limitations. The process is characterized by labor intensity, time consumption, and low fusion efficiency. Moreover, hybridoma clones exhibit genetic instability, which complicates long-term cryopreservation and recovery. From a therapeutic standpoint, the murine origin of these mAbs often limits their clinical applicability in other species due to short half-lives and the potential to induce immunogenic responses (17). Consequently, while hybridoma technology remains a cornerstone of antibody generation, these limitations have necessitated the development of alternative, high-efficiency production platforms.

### 2.2 Phage display technology

Phage display technology enables high-throughput screening of antibody fragments (e.g., single-chain variable fragments, fragments antigen-binding, variable domain of heavy-chain-only antibodies) by fusing their encoding genes with bacteriophage coat proteins, followed by biopanning (Figure 1B) (18). It allows library sizes of 10^10^ to10^12^ variants, far exceeding hybridoma capacity, and has enabled approved human therapeutics such as adalimumab and atezolizumab (19). In veterinary science, phage-derived fragments are being explored for diagnostics and therapy in cattle, poultry, sheep, and swine (20). Despite its versatility, phage display technology faces a key limitation: unstable surface expression of immunoglobulins and their fragments due to the specific structure, which leads to low expression levels or improper folding in the bacterial host (21). Consequently, full-length IgG display remains difficult due to these stability issues. Moreover, amplification bias during selection rounds allows fast-replicating phage clones to outcompete slower-growing clones with higher binding affinity (22). This biological bias can lead to the inadvertent loss of high-affinity binders while disproportionately enriching for clones with robust growth characteristics. Collectively, these factors can constrain the functional diversity and quality of the selected antibody pool, necessitating continued technical optimization.

### 2.3 Single B cell antibody technology

Single B cell antibody technology has emerged as a transformative strategy in the landscape of monoclonal antibody discovery, fundamentally redefining the speed and precision with which high-affinity therapeutics are generated. Unlike traditional hybridoma techniques, which rely on inefficient cell fusion, or phage display platforms that involve the artificial combinatorial pairing of heavy and light chains, this methodology enables the direct interrogation and retrieval of native antibody sequences from individual antigen-specific B lymphocytes (23). The operational workflow integrates advanced high-throughput cell sorting technologies-primarily FACS and microfluidic-based platforms-to isolate single B cells based on specific surface markers or antigen-binding characteristics. Following isolation, single-cell reverse transcription-polymerase chain reaction (RT-PCR) is employed to amplify the variable regions of the VH and VL chains, which are subsequently cloned into expression vectors for recombinant production (Figure 1C) (24). The critical advantage of this approach lies in its preservation of the cognate VH-VL pairing, ensuring that the resulting antibodies retain the natural affinity, specificity, and somatic hypermutation profiles matured *in vivo*. Furthermore, single B cell technology significantly circumvents the extensive screening periods associated with cellular immortalization and avoids the library bias inherent to display technologies, thereby compressing the discovery timeline from months to weeks. This efficiency is particularly advantageous for capturing rare or ultra-potent antibody clones that might be lost during the low-efficiency fusion process of hybridoma generation or outcompeted in bacterial culture systems (25). Consequently, given its ability to rapidly sample the entire immune repertoire, single B cell cloning has become the premier platform for developing neutralizing antibodies against emerging infectious threats and complex pathological targets.

## 3 Process of single B cell antibody discovery

### 3.1 Animal immunization and B cell enrichment

B lymphocytes are typically harvested from the immunological repertoire of host animals, primarily utilizing splenic tissue or peripheral blood. In small animal models, such as mice, the limited volume of peripheral blood necessitates the use of the spleen or lymph node as the primary source for B cell isolation. Splenocytes are obtained via mechanical homogenization, followed by the enrichment of B cells using density gradient centrifugation (26). Conversely, in humans and larger animal species, the abundance of peripheral blood permits the repeated, minimally invasive collection of peripheral blood mononuclear cells (PBMCs), from which antigen-specific B cells are subsequently isolated (27). To enhance the efficiency of antigen-specific B cell isolation, animals typically undergo a multi-dose immunization regimen designed to boost humoral immune responses. Furthermore, heterologous prime-boost strategies utilizing variant vaccine subtypes are often employed to elicit broad-spectrum neutralizing antibodies. In human cohorts, where experimental immunization is constrained by ethical and safety protocols, PBMCs are generally sourced from vaccinated volunteers or convalescent patients to capture naturally developed immunity (28). Prior to cell sorting, serum antibody titers are quantified via ELISA and virus neutralization assays; only individual animals exhibiting high serological titers are selected for downstream B cell isolation.

### 3.2 Identification and isolation of single B cell

The B cell compartment comprises distinct developmental subsets characterized by varying functional roles and antibody secretion capabilities. Class-switched memory B cells and antibody-secreting cells (ASCs), particularly plasmablasts and plasma cells, are the preferred targets for the discovery of high-affinity antibodies. This preference stems from their expression of somatically hypermutated B cell receptors (BCRs) with matured affinity (29). Multiparameter FACS is the conventional method for isolating antigen-specific B cells from total lymphocytes. This process utilizes a panel of fluorochrome-conjugated monoclonal antibodies targeting specific lineage and surface markers, such as IgG, IgM, CD20, and CD19, to discriminate B cell populations (30–32). Crucial to this workflow is the use of labeled “bait” antigens, which must retain the native conformational epitopes of the pathogen to ensure specificity. For example, in SARS-CoV-2 therapeutic discovery, the spike (S) protein receptor-binding domain (RBD) is frequently utilized as a molecular probe to capture potent neutralizing clones (33). In a conventional workflow, FACS is employed to deposit individual cells into single wells, utilizing dual-labeling strategies (antigen positive and lineage positive) to facilitate the precise positive selection of antigen-specific cells while excluding non-specific binders.

### 3.3 Amplification and cloning of Ig genes

**Figure 2:**
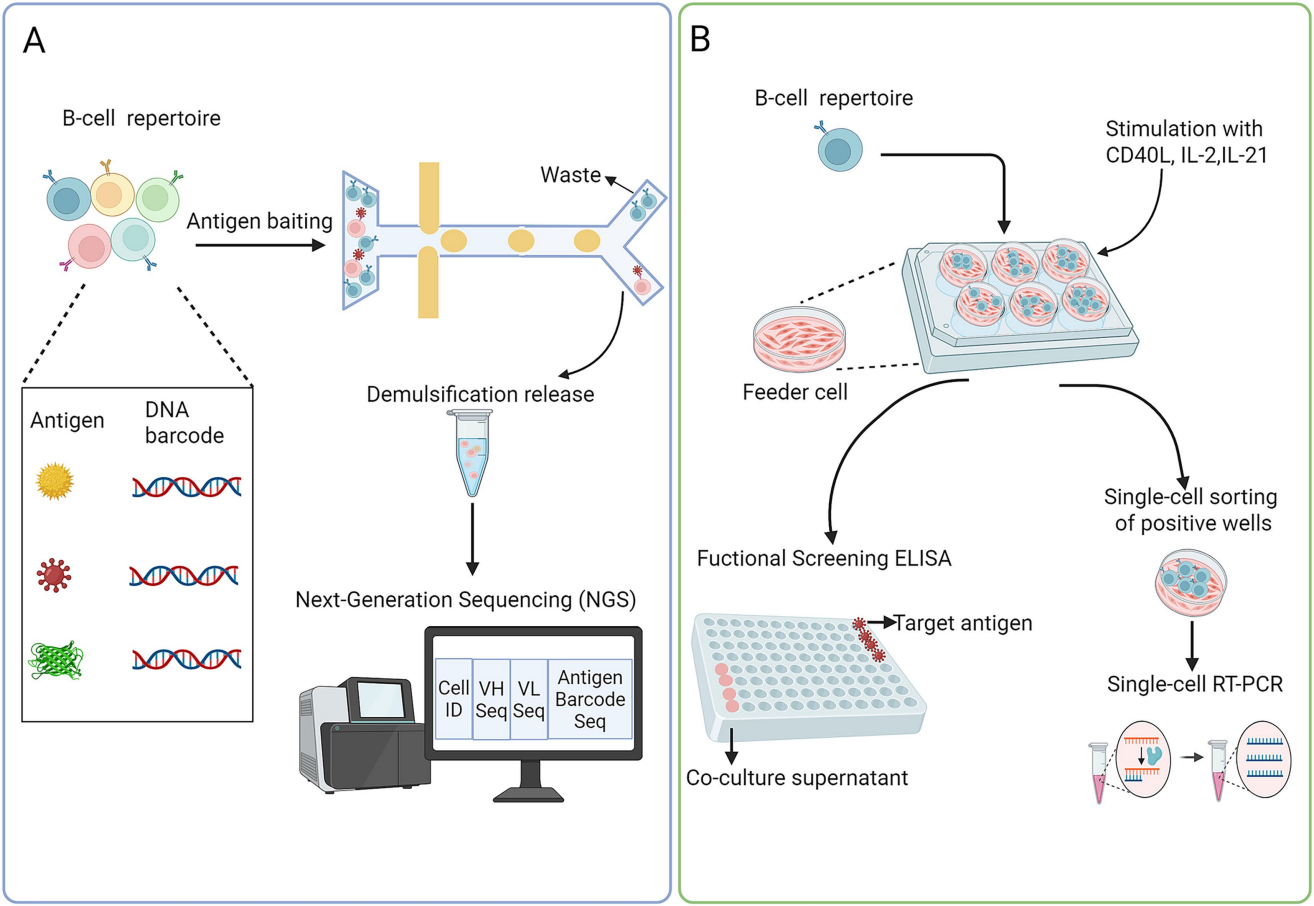
Advanced screening strategles for B-cell selection. **(A)** DNA-barcoding methods (e.g., LIBRA-seq). **(B)** The “screen-first” approach (e.g., Feeder Cell Co-Culture).

Upon isolation, single B cells are deposited directly into 96-well plates containing lysis buffer to liberate intracellular RNA. Due to the minute quantity of RNA recoverable from a single cell, the 96-well format is optimized for seamless transitions to reverse transcription-polymerase chain reaction (RT-PCR) and nested PCR, thereby minimizing sample loss and cross-contamination risks (34). Nested PCR was performed with cDNA from single B cell as the template for the first round of amplification. The products of the first round were then used as templates for the second round of PCR, in which the heavy and light chain variable region genes were amplified using specific primers. Primers were typically designed based on sequences in the IMGT database, with the forward primer located at the signal peptide region of the variable region and the reverse primer located in the CH1 or CL region, where the nucleotide sequences are relatively conserved. Recently, next-generation sequencing (NGS)-based platforms, such as linking B-cell receptor to antigen specificity through sequencing (LIBRA-seq), have gained prominence for the high-throughput retrieval of paired heavy and light chain sequences. LIBRA-seq represents a significant technological leap, enabling the simultaneous interrogation of B cell repertoires against multiple distinct antigen targets (35). By physically coupling antibody sequence data with antigen specificity through DNA-barcoded antigens, this method allows for the screening of millions of single B cells against a diverse epitope landscape, bypassing the spectral overlap limitations inherent in fluorescence-based sorting (Figure 2A). This technology has been successfully applied to identify broad-spectrum antibodies against pathogens such as HIV and influenza virus (36). However, while LIBRA-seq is increasingly utilized in human infectious disease research, its adoption in veterinary medicine remains limited, largely due to the technical complexity and substantial costs associated with the platform.

### 3.4 Evaluation of antibody reactivity and functionality

The production of recombinant antibodies relies on a diverse array of expression platforms, including prokaryotic, plant-based, and mammalian systems. While prokaryotic hosts such as *Escherichia coli* are capable of expressing antibody genes as Fabs, they generally lack the cellular machinery required for essential post-translational modifications and complex protein folding (37). Alternatively, plant-based systems offer an attractive, cost-effective option. However, their widespread adoption is currently constrained by challenges regarding large-scale purification and regulatory safety profiles (38). Consequently, mammalian cell systems remain the industry standard, distinguishing themselves by their ability to ensure correct folding, assembly, and secretion of full-length immunoglobulins. Among these, Chinese hamster ovary (CHO) cells and human embryonic kidney (HEK293) cells are the most prevalent, with CHO cell-derived systems accounting for approximately 70% of all marketed therapeutic antibodies (39). Following expression, antibodies are typically isolated via affinity chromatography, after which their biological activity is characterized using Western Blot (WB), enzyme-linked immunosorbent assay (ELISA), immunofluorescence assays (IFA), and viral neutralization assays.

Conventional discovery workflows, such as nested PCR or LIBRA-seq, typically require the individual cloning, expression, and purification of every identified sequence to determine specificity. This “sequence-first” approach is often labor-intensive and inefficient, as it necessitates the processing of a vast number of clones, many of which may exhibit low affinity or lack functional relevance. To circumvent this bottleneck, Rachael et al. developed a cost-effective feeder cell system stably expressing CD40 ligand (CD40L), interleukin-2 (IL-2), and interleukin-21 (IL-21) (40–42). This platform supports the co-culture of single antigen-specific B cells, promoting their sustained proliferation and continuous antibody secretion *in vitro* (Figure 2B). A critical advantage of this “screen-first” strategy is that it enables the selective amplification and cloning of immunoglobulin genes exclusively from culture wells that demonstrate confirmed antigen reactivity, thereby streamlining downstream characterization. Efforts have been made to develop technical platforms integrating high-throughput with automated culture and screening steps, thus reducing screening time and workload. The Beacon Optofluidic system represents one such optimized approach, which has proven effective in the high-efficiency screening of neutralizing antibodies against human viral pathogens, including respiratory syncytial virus (RSV) and HIV-1 (43–45). This optimized methodology has recently shown significant promise in veterinary applications. Notably, a recent study reported the successful expansion of canine B cells utilizing IL-2 and IL-21 co-stimulation *in vitro* (46). This approach facilitated the precise identification of monoclonal antibodies targeting canine parvovirus (CPV), establishing a valuable technical foundation for the development of novel veterinary therapeutics.

## 4 Applications of single B cell antibody technologies targeting viral diseases in livestock production

### 4.1 Swine

#### 4.1.1 Classical swine fever virus

Classical swine fever (CSF), also known as hog cholera, is a highly contagious disease affecting both domestic and wild swine. It manifests clinically through high fever, anorexia, lethargy, and significant mortality (47–49). Utilizing single B cell isolation, researchers identified three porcine-derived neutralizing mAbs (mAb1, mAb9, and mAb11) targeting CSFV (50). Pigs were immunized with the KNB-E2 subunit vaccine containing the C-strain E2 protein, after which E2-specific B cells were isolated via FACS. When tested against the CSFV Alfort strain, all three mAbs conferred 100% protection to ST cells at a concentration of 125 μg/mL. These porcine-derived mAbs represent promising candidates for the development of passive immunotherapies with reduced immunogenicity and extended half-lives, offering valuable tools for CSF control in both neonatal and adult swine. Other groups isolated mAbs, such as 3A9 and 4F7, by harvesting single B cells from the splenocytes of E2-immunized mice (51). Epitope mapping revealed that these antibodies bind to specific linear B cell epitopes (^25^GLTTTWKEYSHDLQL^39^ and ^259^GNTTVKVHASDERGP^273^, respectively). To enhance the precision of antibody discovery, Dong et al. employed a bait-based flow cytometry strategy using a fluoresceinated linear neutralizing epitope (CTAVSPTTLRTEVVK) combined with FITC-labeled goat anti-pig IgG (52). This approach, applied to pigs pre-screened for high CSFV specific antibody titers, yielded two mAbs (HK24 and HK44) that recognize a conserved linear epitope on the E2 protein. Both antibodies demonstrated high diagnostic specificity and potent neutralizing activity. While sorting with defined epitopes increases selection efficiency, it necessitates prior knowledge of antigenic structures. A potential limitation is that the resulting antibodies target a restricted set of epitopes.

#### 4.1.2 Porcine reproductive and respiratory syndrome virus

Porcine reproductive and respiratory syndrome (PRRS) is a critical infectious disease caused by the PRRSV, causing economic losses globally. In pregnant sows, PRRSV infection leads to severe reproductive failure, including abortion, premature delivery, stillbirths, and mummification. In nursery and fattening pigs, the disease primarily manifests as respiratory distress, such as coughing and dyspnea (53). The viral nonstructural protein nsp12 is a membrane-associated protein whose antigenic properties and functions remain largely uncharacterized (54). Duan et al. addressed this gap by immunizing mice with eukaryotic-expressed nsp12 and utilizing FACS to isolate two mAbs, mAb 1 N14 and mAb 2S18 (55). Through this process, a novel linear epitope (^104^YEFTGNGEDW^113^) was identified using mAb 1 N14. Cellular confocal analysis using the monoclonal antibody mAb 1 N14 enabled the characterization of PRRSV nsp12, which exhibited a distinct punctate distribution throughout the cytoplasm and accumulated markedly in the perinuclear region during early infection. The characterization of nsp12 antigenicity and epitope mapping provides essential insights into the protein’s structure and function. In a valuable study, the isolation of PRRSV neutralizing mAb was successfully achieved using a reverse vaccinology approach (56). Memory B cells were obtained from pigs that had been sequentially exposed to a modified-live PRRSV-2 vaccine and divergent field isolates, followed by B-cell immortalization. Five PRRSV-specific B-cell populations were identified, all of which displayed broad binding to various PRRSV-2 isolates but not to PRRSV-1. Notably, four of the five populations were specific for the GP5 protein, a major envelope glycoprotein. Among these, supernatants from the GP5-binding BNW7p5c4 clone exhibited neutralizing activity, albeit restricted to the PRRSV-2 ATP strain. This insightful research enriches the understanding of PRRSV humoral immunity and serves as a complement to the role of GP5 in neutralization.

#### 4.1.3 Porcine epidemic diarrhea virus

PEDV, a member of the genus Alphacoronavirus, is the etiological agent of porcine epidemic diarrhea (PED). This acute, highly contagious enteric disease is clinically characterized by severe vomiting, watery diarrhea, and rapid dehydration, which result in extensive intestinal villous atrophy and malabsorption. The virus poses a catastrophic threat to the global swine industry, capable of infecting pigs across all demographics but causing up to 100% morbidity and devastating mortality rates in immunologically naive neonatal piglets (57–59). Despite the availability of immunization protocols, current control measures remain insufficient. Extensive research indicates that the classical attenuated vaccine strain, CV777, offers inadequate cross-protection against emerging, highly virulent field variants. Furthermore, maternal antibodies elicited by traditional inactivated vaccines frequently fail to provide sterilizing immunity or sufficient passive protection to piglets during the critical neonatal window (60). Consequently, the generation of mAbs targeting key viral structural proteins has emerged as a pivotal strategy for developing precise diagnostic reagents and effective passive immunotherapeutics. Recent advances in single B-cell sorting technology have significantly accelerated this discovery process. In one notable study, researchers utilized a multi-antigen approach—employing intact PEDV virions, the nucleocapsid (N) protein, and truncated spike (S) proteins as bait to isolate B cells from immunized swine (61). This screening strategy yielded 20 PEDV-specific mAbs, including three N-protein-specific clones that recognized conserved linear epitopes, which are invaluable for developing broad-spectrum diagnostic assays. Crucially, targeting the Spike protein, particularly the S1 subunit responsible for host receptor binding, is essential for virus neutralization. By utilizing eukaryotic-expressed and purified PEDV S1 protein as a screening bait, another study successfully isolated a high-affinity, fully porcine-derived mAb designated C62 (62). Unlike heterologous antibodies, this species-specific mAb minimizes the risk of anti-drug antibody responses. *In vivo* evaluations demonstrated that C62 significantly delayed the onset of clinical symptoms and reduced mortality in challenged piglets, validating the efficacy of single B-cell-derived therapeutics in bridging the protection gap left by conventional vaccines.

#### 4.1.4 African swine fever virus

The control of African swine fever (ASF) is currently limited by the absence of commercial vaccines, necessitating the development of advanced diagnostic tools and therapeutic agents (63). The single B cell antibody technology was recently utilized to target the ASFV CD2v protein, a transmembrane glycoprotein encoded by the EP402R gene, which is critical for hemocyte adsorption, viral immune escape, and serotype classification. In a study leveraging this platform, researchers successfully cloned and expressed antibody genes to generate 14 murine-derived mAbs using recombinant CD2v as the immunogen (64). Validation assays revealed that all 14 mAbs showed reactivity in indirect ELISA, eight of which specifically recognized CD2v in ASFV-infected Porcine Alveolar Macrophages (PAM) cells by IFA. This result demonstrates the capability of the method to generate antibodies that effectively bind to the viral antigen within a cellular context. Furthermore, the study established a blocking ELISA using the specific mAb C89, which demonstrated high specificity, sensitivity, and reproducibility. This application confirms that single B-cell technology provides a robust pipeline for generating high-quality reagents essential for the rapid clinical detection of ASFV and epidemiological monitoring, offering significant advantages over traditional immunogenic methods.

#### 4.1.5 Senecavirus a

In addition to classical swine viral epidemics, researchers have also applied single B-cell antibody technology to emerging viral pathogens. SVA is an emerging pathogen causing vesicular lesions in swine that are clinically indistinguishable from foot-and-mouth disease, necessitating highly accurate differential diagnostics (65). Addressing thelimitations of traditional antibody generation, Ma et al. applied single B-cell sorting to isolate antibodies directly from the natural host species (66). By combining single-cell sorting with reverse transcription-PCR, the researchers isolated B-cells exhibiting specific antigen recognition and amplified the cognate immunoglobulin light- and heavy-chain variable region genes. This process allowed for the production of two whole-porcine monoclonal antibodies, 1 M5 and 1 M25, which maintained the original heavy and light chain pairing naturally occurring in swine. These experimentally derived antibodies were subsequently utilized to engineer a competitive enzyme-linked immunosorbent assay (NAC-ELISA) for the detection of neutralizing antibodies. For detection, 1 M5 functioned as the capture antibody, while biotinylated 1 M25 served as the detection antibody. The resulting diagnostic tool exhibited exceptional performance metrics, boasting a sensitivity of 98.11% and a specificity of 100%. Furthermore, the assay showed strong agreement with the virus neutralization test, which is considered the gold standard, while eliminating cross-reactivity with other virus-infected sera. The “natural host” reagents offer superior specificity for serological surveillance, proving that this technology is not only a research tool but a diagnostic reagent in the practical control and prevention of economically devastating livestock diseases.

### 4.2 Cattle

#### 4.2.1 Foot-and-mouth disease virus

Cattle constitute a cornerstone of the global agricultural economy, yet their productivity is persistently threatened by high-impact pathogens, most notably the FMDV. FMDV serotype O, in particular, remains a prevalent threat to animal husbandry in regions such as China (67). Beyond their agricultural significance, cattle possess a humoral immune system characterized by antibody architectures that are distinct among jawed vertebrates. While typical mammalian antibodies utilize a complementarity-determining region H3 (CDR H3) of approximately 12–16 amino acids, the bovine repertoire is distinguished by an exceptionally long average CDR H3 length of 26 residues. Additionally, the bovine B-cell repertoire exhibits a profound bias in light chain usage, with Igλ comprising approximately 95% of the expressed repertoire, whereas Igκ represents a mere 5% (68–70). The functional significance of these structural anomalies is critical for antiviral defense. Recent studies utilizing single B-cell technologies have successfully isolated 13 neutralizing monoclonal antibodies from cattle infected with FMDV serotype O (71). Statistical correlation from these investigations suggests that antibodies possessing extended CDR H3 regions exhibit superior neutralizing potency. This implies that the unique length of bovine CDR H3 loops plays a vital role in recognizing and neutralizing diverse viral antigens, likely by penetrating the glycan shield or deeply recessed receptor-binding sites on the FMDV capsid.

#### 4.2.2 Bovine viral diarrhea virus

To interrogate the genetic diversity of bovine antibodies specifically the “ultralong” CDR H3 subset often implicated in neutralizing complex viruses like BVDV researchers employ sophisticated single B-cell isolation strategies. These ultralong antibodies feature hypervariable regions extending to an average of 61–62 amino acids, often forming a “knob-on-stalk” motif stabilized by diverse disulfide bonds (72). To capture these sequences, flow cytometry-based sorting is typically coupled with various downstream platforms: (1) low-throughput limited-cycle semi-nested PCR with Sanger sequencing; (2) medium-throughput PCR amplification with indexing for Next-Generation Sequencing (NGS); and (3) high-throughput 10x Chromium Next Gel Bead-in-Emulsion (GEM) processing. A comparative assessment by Ramirez Valdez et al. utilizing the Ig-Sequence Multi-Species Annotation Tool demonstrated high concordance across these platforms, validating their utility for repertoire analysis (73). The application of the methodology has proven essential in studying BVDV. By collecting lymphocytes from a BVDV-immunized cow, researchers successfully isolated an ultralong CDR H3 antibody, designated clone H12 (74). This specific antibody demonstrated potent, dose-dependent viral binding. The isolation of H12 underscores the structural-functional relationship of bovine antibodies, highlighting how the evolutionary adaptation of ultralong CDR H3 regions facilitates the binding of difficult viral targets such as BVDV.

## 5 Advanced antibody discovery for viral diseases management in companion animals

### 5.1 Dogs

#### 5.1.1 Canine distemper virus

CDV persists as a devastating pathogen in carnivores, with control measures increasingly compromised by vaccine-breakthrough infections attributed to genetic divergence and immunization waning (75). While passive immunotherapy presents a viable post-exposure intervention, traditional production methods utilizing murine hybridomas or phage display often yield reagents with limited clinical utility. To circumvent the challenges of allergic reactions and decreased half-life, researchers leveraged single B-cell sorting to mine the native canine immune repertoire. By isolating individual B-cells via FACS and amplifying native heavy and light chain genes, researchers generated fully canine mAbs that ensure natural host pairing and biocompatibility (76). Notably, this approach identified mAb D16, a high-affinity candidate targeting the CDV hemagglutinin (H) protein. *In vivo* therapeutic trials, D16 administration provided effective protection against lethal challenge, evidenced by statistically significant improvements in survival, marked reductions in systemic viral loads, and the alleviation of severe pulmonary pathology such as interstitial pneumonia. This success underscores the capacity of single B-cell technology to produce antibodies that maintain the structural integrity and effector functions necessary for viral clearance without the risk of heterologous immunogenicity. Although the high mutation of RNA viruses suggests that therapeutic cocktails targeting non-overlapping epitopes may eventually be required to prevent escape mutants, the rapid generation of potent, species-specific candidates like D16 confirms this technology as a robust platform for developing next-generation veterinary antivirals.

#### 5.1.2 Canine parvovirus

CPV remains a highly virulent pathogen responsible for acute hemorrhagic enteritis and myocarditis, particularly in distinctively vulnerable neonatal and juvenile canine populations (77). While vaccination constitutes the cornerstone of prophylaxis, the “immunity gap”-wherein maternally derived antibodies interfere with active immunization without providing complete protection-frequently precipitates vaccine failure and subsequent infection (78–80). Consequently, the development of therapeutic native canine neutralizing antibodies has emerged as a crucial need in veterinary medicine. Addressing this need, recent investigations have leveraged single B-cell sorting to mine the host immune repertoire. In one foundational study, researchers utilized FACS to isolate specific IgM^−^/IgG^+^ B cells from peripheral blood (81). By employing nested PCR to amplify variable region genes (VH and VL) and expressing the resulting constructs in HEK293 cells, the team generated 60 mAbs, five of which exhibited potent neutralizing activity. The necessity for such agile discovery platforms is underscored by the virus’s rapid evolutionary trajectory. CPV is characterized by a high mutation rate, which has driven the emergence of diverse antigenic variants, including CPV-2a, CPV-2b, CPV-2c, and their subsequent new lineages (82). Single B-cell technology is uniquely positioned to address this antigenic drift due to its speed and specificity. Recognizing the need for greater efficiency, Fruh et al. introduced a methodological optimization to the standard workflow. Their protocol combined flow cytometric sorting using fluorescent virus-like particles (VLPs) as “bait” with a subsequent B-cell culture step (46). Rather than blindly sequencing all sorted cells, they screened culture supernatants for specific immunoglobulin binding prior to genetic amplification. This functional interrogation allowed for the selective identification of high-affinity clones before the labor-intensive steps of sequence amplification and vector construction. By verifying capsid binding via ELISA early in the pipeline, this refined approach effectively filters out low-affinity binders. Ultimately, this streamlining significantly reduces the development timeline and economic burden associated with the purification of non-neutralizing antibodies, marking the advancement in the production of high-value veterinary therapeutics.

### 5.2 Equids

#### 5.2.1 Equine influenza virus

Equine influenza is an acute, highly contagious respiratory disease caused by the equine influenza A virus that affects all equine species. It is a listed disease by the World Organization for Animal Health (WOAH) and causes acute respiratory infection, with typical clinical signs including high fever, dry cough, purulent nasal discharge, and loss of appetite (83). Lin et al. established a platform utilizing EIV hemagglutinin (HA) protein as an antigenic model to isolate neutralizing mAbs against different EIV strains from single B cells within equine BCR reservoirs (84). Among the resulting antibodies, one clone, designated H81, demonstrated superior cross-neutralizing activity against multiple EIV strains *in vitro*. Furthermore, it effectively protected EIV-challenged mice, resulting in significantly improved survival, reduced pulmonary inflammation and decreased viral titers. Through the structural analysis, a conserved functional region comprising 27 key amino acids was identified within the HA protein across circulating EIV strains. Notably, 12 residues within this region showed the high predicted binding affinity to H81. Importantly, the predicted epitope overlapped with the known receptor-binding site of equine HA, providing a mechanistic explanation for its broad neutralization capacity. This work developed a method for profiling the equine B-cell receptor repertoire following infection or vaccination.

## 6 Animal immune repertoires targeting zoonotic pathogens

### 6.1 Nipah virus

The utility of single B-cell antibody technology in addressing emerging zoonotic threats is illustrated by recent advancements in the diagnostics of Nipah virus (NiV), a BSL-4 pathogen with high case fatality rates (85). Traditional antibody discovery methods, such as hybridoma technology, often struggle to meet the urgent demands of outbreak scenarios due to low fusion efficiencies and the potential loss of natural heavy-light chain pairing. To overcome these bottlenecks, recent research employed high-throughput single B-cell screening via multicolor FACS to interrogate the immune repertoire of immunized mice (86). This approach facilitated the direct and rapid isolation of five high-affinity monoclonal antibodies targeting the NiV glycoprotein (G), the immunodominant surface protein responsible for host cell invasion. By circumventing the constraints of random fusion, this technology ensured the preservation of native antibody specificity and affinity, which were subsequently leveraged to construct a novel Amplified Luminescent Proximity Homogeneous Assay (AlphaLISA). The resulting diagnostic platform demonstrated superior performance metrics compared to traditional serological methods, achieving a detection sensitivity approximately 41.7 times higher than conventional ELISA. Furthermore, the assay significantly reduced operational complexity and turnaround time to under 30 min while maintaining robust specificity against other zoonotic viruses such as Zika and Japanese encephalitis. This application underscores the critical role of single B-cell technology in accelerating the development of next-generation diagnostic tools that are essential for early detection and surveillance in underserved regions prone to zoonotic spillover.

### 6.2 Human immunodeficiency virus

The expansion of single B-cell antibody technology beyond traditional murine models is transforming the immune repertoires for zoonotic research and comparative immunology. For example, rhesus macaques serve as essential surrogates for human vaccine efficacy, yet the tools to dissect their humoral responses have historically lagged behind. Addressing this, researchers recently optimized a flow cytometry-based strategy to isolate single HIV-1 envelope specific B cells from macaques (87). By employing fluorochrome-labeled antigens and strategically omitting anti-IgG antibodies during sorting to prevent interference, this protocol enables the precise cloning of high-affinity macaque monoclonal antibodies. This advancement allows for a granular analysis of vaccine-induced immunity in non-human primates that closely mirrors established human methodologies.

Simultaneously, single B-cell technologies allow researchers to exploit the unique evolutionary biology of veterinary species to target pathogens that evade human immune responses. A striking application involves the immunization of cows to generate broadly neutralizing antibodies (bnAbs) against HIV (88). Unlike human or rodent antibodies, bovine antibodies possess an ultralong third heavy chain complementary determining region (HCDR3), which can exceed 70 amino acids. Leveraging this structural anomaly, researchers demonstrated that cows immunized with the BG505 SOSIP trimer developed broad serum neutralization in as little as 42 days-a process that typically takes years in humans (89). Through single-cell isolation, a monoclonal antibody with a 60-amino acid HCDR3 was identified, capable of penetrating occluded epitopes on the viral envelope and neutralizing 72% of cross-clade isolates. Collectively, these studies underscore the power of single B-cell platforms to not only refine diagnostics and vaccine assessment in primate models but also to mine the distinct antibody repertoires of livestock for novel therapeutic agents against complex infectious diseases.

### 6.3 Mpox virus

The recent global outbreak of mpox, a zoonotic infection caused by the MPXV, highlights the critical need for rapid diagnostic tools that can function independently of complex laboratory infrastructure (90–92). Recent research has demonstrated the efficacy of combining mRNA immunization with high-throughput single B-cell sequencing. In an application of this pipeline, researchers successfully targeted the A29L surface protein of MPXV to generate high-affinity mAbs (93). By immunizing mice directly with mRNA encoding A29L rather than recombinant proteins, the study circumvented complex purification steps while inducing a robust humoral response. Subsequently, high-throughput V(D)J region sequencing allowed for the comprehensive lineage analysis of B cell, facilitating the identification of eight specific antibody candidates. This approach represents a marked improvement over traditional hybridoma techniques, which are often hindered by low efficiency and the loss of rare, high-affinity clones during the fusion process. The practical utility of these single B-cell-derived antibodies was validated through the development of an ultrasensitive fluorescent immunochromatographic assay (SiTQD-ICA). Utilizing the identified M53 and M78 antibodies, this biosensor achieved a minimum detection limit of 5 pg./mL within 20 min, maintaining high specificity against clinical differentials such as chickenpox and various respiratory pathogens. This capability is particularly pertinent for zoonotic surveillance, as it enables the detection of viral antigens in pharyngeal swabs prior to the onset of characteristic rashes, thereby addressing the challenge of hidden transmission during the incubation period. By significantly shortening the timeline from antigen introduction to antibody production, this methodology offers a scalable and versatile framework for responding to future zoonotic spillovers.

### 6.4 Rift Valley fever virus

RVFV poses a dual threat to livestock and humans, capable of causing fatal hemorrhagic fever and encephalitis (94). The urgent need for therapeutic interventions against RVFV, a mosquito-borne zoonotic phlebovirus with pandemic potential. With no licensed human vaccines currently available, the development of neutralizing mAbs targeting the viral surface glycoproteins Gn and Gc-critical for viral attachment and fusion-has become a priority (95). Recent research has successfully demonstrated the efficacy of high-throughput single lymphocyte transcriptomics using the 10x Genomics platform. In this approach, researchers streamlined the discovery pipeline by integrating flow cytometric sorting with droplet-based single-cell RNA sequencing (scRNA-seq) (96). Following the immunization of mice with the live-attenuated rMP-12 strain and subsequent protein boosts, memory B cell populations were isolated based on antigen specificity. A key advantage of this methodology is the preservation of the natural affinity and specificity during the sequencing of the antibody repertoire. This high-resolution workflow facilitated the rapid selection and recombinant expression of 23 candidate mAbs. Subsequent characterization revealed that approximately half of these candidates exhibited high-affinity specific binding to their cognate antigens. This study not only yielded potential therapeutic candidates but also validated the use of single B cell transcriptomics as a robust, high-throughput engine for accelerating antibody discovery against complex zoonotic viral targets.

## 7 Prospects of single B cell antibody technology in veterinary medicine

The landscape of veterinary immunology is undergoing a transformative shift as single B cell antibody discovery platforms increasingly replace traditional methodologies. The research and application of this technology mentioned in this article have been summarized in Table 1. Historically, the development of mAbs for animal health was restricted by the technical limitations of hybridoma technology and the high costs associated with recombinant protein engineering. This section explores the technical advantages, unique biological opportunities, and clinical potential of single B cell technology in the context of animal health.

**Table 1:** Summary of the single B Cell derived antibodies against veterinary viruses.

Virus	Host species	Antigen bait	Application of antibody	Ref.
Classical swine fever virus (CSFV)	Swine	E2 protein	Therapy (*vitro*)	(47)
Classical swine fever virus (CSFV)	Swine	E2 protein	Diagnosis (blocking ELISA)	(48)
Classical swine fever virus (CSFV)	Swine	Epitope (^828^CTAVSPTTLRTEVVK^842^)	Therapy (*vitro*)	(49)
Porcine reproductive and respiratory syndrome virus (PRRSV)	Swine	Nsp12	Epitope identification	(52)
Porcine epidemic diarrhea virus (PEDV)	Swine	PEDV; S1 protein; N protein	Experimental research	(57)
Porcine epidemic diarrhea virus (PEDV)	Swine	S1 protein	Therapy (piglet)	(58)
African swine fever virus (ASFV)	Swine	Recombinant CD2v protein	Diagnosis (blocking ELISA)	(60)
Senecavirus A (SVA)	Swine	Senecavirus A	Diagnosis (ELISA for the detection of neutralizing antibodies)	(62)
Foot-and-mouth disease virus (FMDV)	Bovine	FMDV	Experimental research	(67)
Bovine viral diarrhea virus (BVDV)	Bovine	BVDV	Experimental research	(70)
Canine distemper virus (CDV)	Canine	Hemagglutinin protein	Therapy (dog)	(74)
Canine parvovirus (CPV)	Canine	CPV	Experimental research	(79)
Canine parvovirus (CPV)	Canine	Virus-Like Particles (VLPs)	Therapy (*vitro*)	(43)
Equine influenza virus (EIV)	Equids	Hemagglutinin protein	Therapy (mouse)	(82)
Nipah virus (NiV)	Murine (Model)	Glycoprotein	Diagnosis (amplified luminescent proximity homogeneous assay)	(84)
Human immunodeficiency virus (HIV)	Rhesus macaque (Model)	Envelope glycoprotein	Experimental research	(85)
Human immunodeficiency virus (HIV)	Bovine (Model)	Envelope glycoprotein	Experimental research	(86)
Monkeypox virus (MPXV)	Murine (Model)	A29L protein	Diagnosis (fluorescent immunochromatographic assay)	(91)
Rift valley fever virus (RVFV)	Murine (Model)	Gn and Gc glycoproteins	Experimental research	(94)

The primary advantage of single B cell technology lies in the high speed and efficiency with which lead antibody candidates can be isolated. Traditional hybridoma technology, which has served as the gold standard for decades, is frequently hampered by the stochastic nature of somatic cell fusion. The efficiency of fusion between B cells and myeloma partners is notoriously low, often yielding a limited number of viable clones from a large pool of splenocytes. By labeling B cells with fluorescently conjugated antigens, researchers can precisely isolate memory B cells or plasmablasts that exhibit the highest binding affinity. This direct approach eliminates the need for cell immortalization, thereby bypassing the traditional bottlenecks of fusion efficiency and clone instability. Consequently, the transition from animal immunization to the identification of heavy and light chain sequences can be compressed into a timeframe of one to 2 weeks, a critical advantage for responding to emerging zoonotic or epizootic outbreaks. A significant limitation of both hybridoma technology and phage display is the potential loss of immunological diversity. In hybridoma production, the “repertoire bias” occurs because only a fraction of the B cell population successfully fuses and survives selection, often favoring high-abundance clones over rare, high-affinity ones. Similarly, while phage display allows for the screening of massive libraries, the random shuffling of VH and light VL chains during library construction can disrupt the natural cognate pairing found in the host. Single B cell technology preserves the natural pairing by sequestering individual cells before lysis and RT-PCR. This fidelity allows researchers to access the full breadth of the immune repertoire, including rare B cell clones that may possess unique neutralizing capabilities. Furthermore, because the antibodies are sourced directly from the host’s refined immune response, they typically exhibit superior biophysical properties, such as high solubility and low polyreactivity, which are essential for downstream manufacturing and clinical efficacy.

Veterinary species possess unique structural and genetic immunological characteristics that can be uniquely exploited through single B cell sequencing. One of the most striking examples is found in bovine immunology. Cattle are known to produce a subset of antibodies characterized by ultralong CDR H3 loops, which can exceed 70 amino acids in length. These loops form a unique “knob and stalk” structure that extends far from the antibody surface, allowing them to penetrate “cryptic” or recessed epitopes on viral antigens, which are physically inaccessible to standard human or murine antibodies (97). Single B cell technology enables the precise recovery of these complex bovine sequences. These structures are often difficult to maintain or correctly fold in phage display libraries due to their unconventional architecture and reliance on specific disulfide bond patterns. Similarly, the technology has facilitated the discovery of high-affinity rabbit monoclonal antibodies. Rabbit B cells utilize gene conversion to diversify their repertoire, generally resulting in antibodies with higher affinity and specificity than those derived from mice (98). These rabbit-derived reagents are increasingly favored for veterinary diagnostics.

The commercial landscape for veterinary biologics has been fundamentally altered by the success of lokivetmab (Cytopoint®). As a caninized mAb targeting interleukin-31 (IL-31) for the treatment of canine atopic dermatitis, lokivetmab has demonstrated that there is a robust market for high-quality, species-specific antibody therapies (99). The clinical efficacy of this molecule validated the “speciesization” approach, where the constant regions of the antibody are matched to the host species to minimize immunogenicity and maximize serum half-life. The single B cell technology offers a streamlined pathway to develop the next generation of these therapeutics. By isolating B cells directly from convalescent dogs, cats, or horses, researchers can identify lead candidates that already possess the requisite framework sequences for the target species, thereby requiring minimal molecular engineering. This “native” discovery approach is particularly promising in the field of veterinary oncology. Currently, checkpoint inhibitors targeting the PD-1/PD-L1 axis are being investigated for blockade therapy in canine mast cell tumors and melanomas (100–102). The ability to rapidly isolate high-affinity, canine-specific anti-PD-1 antibodies from dogs with spontaneous tumor regression could revolutionize the treatment of cancer in companion animals.

## 8 Challenges of single B cell antibody technology in veterinary medicine

The transition from traditional hybridoma technology to single B cell platform represents a paradigm shift in veterinary immunology, offering the promise of rapid discovery and high-diversity antibody repertoires. However, the translation of these technologies from human medicine to the veterinary sector is fraught with unique biological, technical, and economic hurdles. While the fundamental principles of B cell selection remain constant, the practical application across diverse animal species introduces complexities that are often absent in human or murine studies.

A significant hurdle in veterinary single B cell analysis is the lack of comprehensive genomic data for many species. While human and murine immunoglobulin (Ig) loci are well-mapped, the germline V(D)J repertoires for minor veterinary species (felines, specific avian breeds, or minor livestock) remain incompletely annotated (103–105). The lack of high-quality reference genomes directly impacts the molecular recovery of antibody genes. Single B cell technologies typically rely on the amplification of heavy and light chain variable regions via PCR. Without precise knowledge of the leader sequences or framework 1 (FR1) regions, researchers must resort to degenerate primers (106). This approach often suffers from reduced sensitivity and biased amplification, potentially leading to the loss of high-affinity clones that do not perfectly match the primer consensus. Moreover, successful recovery of single B cells relies heavily on the ability to distinguish and isolate target subsets, such as antigen-specific memory B cells or plasmablasts, through high-parameter FACS. In human medicine, a vast array of validated fluorophore-conjugated antibodies exists to target surface markers like CD19, CD20, and CD38 (107). In the veterinary field, there is a chronic shortage of such validated, species-specific secondary reagents. In many target species, definitive surface markers that distinguish memory B cells or plasma cells from naive B cells, such as CD27 or CD138 in humans, have yet to be identified, or their specific monoclonal antibodies are not commercially available for veterinary use. Fluorescence-conjugated antibodies against such markers are used to label cells, enabling subsequent cell sorting or phenotypic analysis in FACS. Researchers often attempt to use cross-reactive anti-human or anti-mouse antibodies, but these frequently exhibit low affinity or non-specific binding in veterinary samples (108). This “reagent gap” necessitates time-consuming and expensive preliminary work to validate markers for each new species, significantly slowing the development of mAbs for emerging animal diseases.

When high-affinity antibodies are successfully isolated, their clinical translation is often hindered by immunogenicity. If a mouse-derived or rabbit-derived antibody is administered to a dog or a cat, the host’s immune system recognizes the molecule as foreign. This triggers the production of anti-drug antibodies (ADAs), which can impair the therapeutic effect or cause hypersensitivity reactions (109). To mitigate this, antibodies should be adapted for use in other species through a process analogous to humanization. This engineering is not a simple duplication procedure. The structural integrity of the framework regions is critical for maintaining the native configuration of the CDR loops; its alteration can adversely affect the paratope geometry and lead to a reduction in antigen binding affinity (110). Ensuring that the engineered antibody retains its original biophysical properties requires multiple rounds of modeling and empirical testing.

The economic framework of veterinary medicine differs fundamentally from the human pharmaceutical industry. In human health, the high development costs of mAbs can often be offset by high retail prices, especially for oncology or autoimmune therapies. In contrast, veterinary therapeutics, especially those developed for the livestock sector, must maintain cost-effectiveness to guarantee a sustainable return on investment for producers (111). The high capital expenditure associated with scRNA-seq, microfluidic platforms, and high-throughput recombinant expression remains a significant barrier to entry for veterinary laboratories. While these technologies provide comprehensive screening coverage, the “cost per dose” of the final product remains a critical bottleneck. For a veterinary mAb to be commercially viable, the workflow must be optimized for cost-efficiency without sacrificing the specificity and affinity required for clinical efficacy. Finally, the transition from a laboratory-scale single B cell clone to a large-scale manufactured product introduces rigorous quality control requirements (112). Veterinary biologics must meet stringent safety and purity standards, yet the production facilities often operate on thinner margins than their human-sector counterparts. The stability of antibody is a major concern. Veterinary medicines must often endure substandard cold chain conditions during transport to rural farms or clinics. Developing formulations that preserve the structural integrity of the mAb, which necessitates the prevention of aggregation and denaturation, further complicates the progression of the development timeline (113). The regulatory pathways for veterinary biologics, while distinct from human pharmaceuticals, still require extensive safety data and field trials.

## 9 Conclusion

Single B cell antibody technology stands at the forefront in veterinary medicine, offering the potential to rapidly generate high-affinity, naturally paired antibodies against viral pathogens and chronic diseases. The ability to mine the unique immune repertoires of species like cows and pigs offers novel structural solutions for antigen recognition. However, realizing the full potential of this technology requires overcoming significant hurdles related to genomic annotation, reagent availability, and production costs. Future research should prioritize expanding public Ig germline databases for veterinary species and developing cost-efficient screening platforms to bridge the gap between academic discovery and clinical application.
